# Illness uncertainty, social support, and coping mode in hospitalized patients with systemic lupus erythematosus in a hospital in Shaanxi, China

**DOI:** 10.1371/journal.pone.0211313

**Published:** 2019-02-21

**Authors:** Xin Li, Lan He, Jing Wang, Mingxu Wang

**Affiliations:** 1 The First Affiliated Hospital of Xi’an Jiaotong University, Xi’an, Shaanxi, China; 2 School of Public Health, Health Science Center of Xi’an Jiaotong University, Xi’an, Shaanxi, China; Campus Bio-Medico University of Roma, ITALY

## Abstract

**Objective:**

To analyze the relationships between illness uncertainty, social support, and coping mode in hospitalized patients with systematic lupus erythematosus (SLE).

**Methods:**

The General Health Questionnaire, Mishel Uncertainty in Illness Scale, Social Support Rating Scale, and Medical Coping Modes Questionnaire were to the hospitalized patients with SLE (N = 200) in a tertiary hospital in Shaanxi.

**Results:**

The hospitalized patients with SLE showed a moderate level of illness uncertainty. Furthermore, illness uncertainty was negatively correlated with support availability (*r =* -0.161, *P* = 0.023) and facing coping mode (*r =* -0.231, *P* = 0.001), and was positively correlated with the yielding coping mode (*r =* 0.249, *P* < 0.001).

**Conclusion:**

These findings suggest that support availability and coping modes were associated with moderate level of illness uncertainty, indicating that support availability support should be strengthened in hospitalized patients to actively face their disease. This subsequently improves their treatment compliance and quality of life.

## Introduction

Systemic lupus erythematosus (SLE) is an autoimmune disease of the connective tissue. It is characterized by tissue injury and displays clinical manifestations in multiple systems and organs due to the occurrence of large numbers of pathogenic autoantibodies and immune complexes [1]. SLE presents with a chronic, alternating recurrence and the remission process showed considerable heterogeneity in clinical manifestations and severities, making the patients unable to predict the disease process or prognosis [2]. This in turn leads to a greater illness uncertainty, and is defined as lack of capacity to accurately discern the disease-related events or assess the significance and predict the outcomes of such events [3].

Education level, coping style, social support, and information support are considered the four main factors that influence the illness uncertainty [4]. Awareness of illness uncertainty can influence patients’ emotions, coping modes, psychology, social adaptivity, and other issues [5], as well as their treatment compliance behavior and quality of life [6–7]. Social support can help improve SLE patients’ coping and capacity to adapt with the psychological stress brought on by their disease [8]. At present, research on illness uncertainty involved tumors, heart disease and chronic disease [9–13]. Both domestically and abroad, there are very few studies exploring the illness uncertainty of SLE patients, and all these studies that exist consist of small sample sizes [14–17]. Hence the present study investigated the relationships between illness uncertainty, social support, and coping modes among 200 SLE patients who have been hospitalized for over a week in a tertiary hospital in Shaanxi province, China, providing a theoretical basis for developing intervention measures to lessen illness uncertainty in such patients.

## Materials resource and methods

### Subjects

SLE patients with SLE hospitalized from December 2015 to July 2016 in a tertiary hospital in Shaanxi Province, China, were selected by simple random sampling. This study was approved by the ethics committee of the First Affiliated Hospital of Xi’an, Jiaotong University (approval no. 2015012010). Written informed consent was obtained from all the participants. The criteria used for patient selection and exclusion are shown in Table 1.

**Table 1:** Inclusion and exclusion criteria of SLE patients.

	Inclusion Criteria	Exclusion Criteria
1	conformed to the SLE classification criteria as revised in 1997 by the American College of Rheumatology (ACR)	having disorders of consciousness and communication
2	hospitalized for at least a week	using psychotropic drugs such as antidepressants and tranquilizers
3	being able to communicate and comprehend the questionnaires	having neuropsychiatric SLE
4	provided their written informed consent, or from parents/guardians for minors	having severe dyslexia caused by SLE

### Instruments

The researchers explained the research objective to the participants when handing out the questionnaires. After obtaining the participants’ informed consent, they were asked to complete the questionnaires. For patients with dyslexia, questionnaire items were read out one by one by the researchers, and then recorded their responses. All questionnaires were collected on the spot and checked for effectiveness.

### General characteristics

This questionnaire assessed participants’ age, gender, ethnicity, occupation, educational level, marital status, family relationships, disease duration, self-perceived severity of disease, medical fee payment method, and family income.

### Mishel Uncertainty in Illness Scale (MUIS)

This is a contains 33-item scale and measures the four dimensions of illness uncertainty, including unpredictability, complexity, ambiguity, and lack of information. The 15th item is not attributed to any dimension; and hence, it was not included in the final results. The total score of the MUIS ranges from 32 to 160, and was defined as 3 levels. The range of 32 to 74.7 was defined as low level, 74.8 to 117.4 as moderate level, 117.5 to 160 as high level, and higher scores indicating a higher degree of uncertainty in illness[18]. The Cronbach’s alpha of the scale was 0.865 and the content validity index (CVI) was 0.92 [19].

### Social Support Rating Scale (SSRS)

This is a 10-item scale and is divided into 3 subscales: objective support, subjective support, and social support availability. The total score ranges from 12 to 60, and higher scores indicate better social support [20]. The Cronbach's alpha coefficient of the scale was 0.814.

### Medical Coping Modes Questionnaires (MCMQ)

The questionnaire contained 20 items in 3 subscales: facing, avoiding, and yielding. The Cronbach's alpha coefficient of the scale was 0.69, 0.60 and 0.76. Higher scores indicated that the patient was more likely to choose the coping mode [21].

### Statistical analysis

The software package EpiData 3.1 (EpiData Entry version, Denmark) was employed to conduct double entry verification of the collected data and analyzed with PASW Statistics 18.0. (SPSS Inc., Chicago, IL). Continuous data were expressed as means ± SD ($\bar{x}$ ± s), while categorical data were expressed as numbers and percentages (%).Demographic characteristics were compared between the groups using one-way analysis (ANOVA) of variance and differences within each group were compared using multivariate analysis of variance. The date of two groups were compared using independent sample *t*-test to detect the significance. Linear correlation between the variables was assessed by Spearman correlation test. A significance level of P< 0.01 was considered to be statistically significant in multiple testing and a significance level of P < 0.05 was considered statistically significant in other analysis.

## Results

### General characteristics

During the study period, total of 268 SLE patients were admitted to the hospital. According to the inclusion and exclusion criteria, 236 patients were enrolled in the study. These patients were numbered according to their admission sequence, and 210 hospitalized patients with SLE were randomly selected by using a random number table. A total of 210 questionnaires were distributed among the patients. Seven people signed the informed consent of the questionnaires but refused to fill in the questionnaires were removed from the total number of the study. Of the 203 patients, 3 hospitalized female patients submitted invalid questionnaires as less than 20% of total survey projects were finished. The proportion of missing data in the study was less than 5%. The patients who completed the questionnaires showed no significant differences between the groups in age, disease duration, educational level, marital status, and medical fee payment method.

Majority of the hospitalized patients with SLE were female and from Shaanxi Province. The age of the patients ranged from 13 to 74 years old, and the average age was 38.27±13.51. All the patients belonged to the Han ethnicity. The specific results are shown in Table 2.

**Table 2:** Differences in the MUIS scores across each demographic characteristics among SLE patients*.

Characteristics	SLE Patients (n = 200)	MUIS Scores	P-Value**
**Gender, n(%)**			0.966
** Male**	23 (11.50)	97.19 ± 9.77	
** Female**	177 (88.50)	97.08 ± 11.62	
**Marital Status, n(%)**			0.044
** Unmarried**	47 (23.50)	93.15 ± 12.59	
** Married**	140 (70.00)	98.23 ± 11.00	
** Divorced, separated or widowed**	13 (6.50)	98.60 ± 8.08	
**Educational level, n(%)**			<0.001^§^
** Primary school and below**	18 (9.00)	100.87 ± 7.29	
** Junior middle school**	58 (29.00)	100.62 ± 9.52	
** Senior high school or special middle school**	56 (28.00)	97.33 ± 10.28	
** Junior College**	33 (16.50)	95.46 ± 10.98	
** Bachelor degree or above**	35 (17.50)	90.94 ± 14.72	
**Work status, n(%)**			0.600
** Full-time**	90 (45.00)	96.21 ± 12.84	
** Part-time**	10 (5.00)	96.50 ± 11.91	
** Retired or unemployed**	100 (50.00)	97.99 ± 9.84	
**Family relationship, n(%)**			0.929
** Good**	163 (81.50)	96.96 ± 11.54	
** Common**	32 (16.00)	97.88 ± 11.09	
** Bad**	5 (2.50)	96.75 ± 9.81	
**Disease duration (years), n(%)**			0.292
** <1**	67 (33.50)	98.15 ± 10.98	
** 1–5**	68 (34.00)	97.20 ± 11.58	
** 6–10**	32 (16.00)	93.23 ± 10.77	
** >10**	33 (16.50)	98.10 ± 12.13	
**Self-perceived severity of disease, n(%)**			0.012
** Very severe**	70 (35.00)	100.53 ± 9.54	
** Severe**	101 (50.50)	95.67 ± 11.59	
** Less severe**	29 (14.50)	93.96 ± 13.09	
**Payment of medicine, n(%)**			0.048
** Full or part of public funds/insurance**	8 (4.00)	95.63 ± 5.29	
** Medicare or social security**	94 (47.00)	94.96 ± 12.78	
** NCMS**	74 (37.00)	100.24 ± 9.99	
** Self-financed**	24 (12.00)	96.95 ± 9.01	
**Family income (Yuan/month), n(%)**			0.001^♀^
** ≤1,000**	31 (15.50)	101.78 ± 9.37	
** 1,001–2,000**	55 (27.50)	100.30 ± 10.26	
** 2,001–3,000**	62 (31.00)	96.00 ± 10.41	
** ≥3,001**	52 (26.00)	92.52 ± 12.78	

MUIS, Mishel Uncertainty in Illness Scale; NCMS, New rural Cooperative Medical System.

*Values are expressed as mean ± standard deviation unless otherwise specified.

**P Values for the analysis was performed using one-way ANOVA test and a significance level of *P* < 0.01 was considered statistically significant.

^§^Five groups of educational level of hospitalized SLE patients with uncertainty of illness scores using multivariate analysis of variance of the *P* values was <0.001.

^♀^Four groups of family income of hospitalized SLE patients with uncertainty of illness scores using multivariate analysis of variance of the *P* values was 0.001.

### Illness uncertainty

In 190 hospitalized patients (95%) with SLE, the MUIS scores were greater than 80. The mean score was 97.60 ± 11.24, indicating that majority of the patients had a moderate level uncertainty. The MUIS total and subscale scores are shown in Table 3.

**Table 3:** MUIS Total and Subscale Scores.

Items	Possible range in total score	Score Range	$\bar{x}$ ± s
**Unpredictability**	5–25	7–25	16.07 ± 3.05
**Complexity**	7–35	7–26	16.70 ± 2.88
**Indeterminacy**	13–65	18–59	41.79 ± 7.01
**Lack of information**	7–35	10–32	23.04 ± 4.48
**Total**	32–160	55–121	97.60 ± 11.24

MUIS, Mishel Uncertainty in Illness Scale.

### Comparison of illness uncertainty by demographic characteristics

We examined the influence of demographic characteristics on illness uncertainty using one-way analysis of variance. Results indicated that illness uncertainty in SLE patients differed significantly by educational level (*F =* 4.492, *P* = 0.002), and family income (*F =* 5.978, *P* = 0.001). In order to exclude the potential confounders in demographic factors, the five groups of educational level of hospitalized SLE patients with illness uncertainty scores using multivariate analysis of variance significantly differed (*F =* 5.243, *P*<0.001), and the four groups of family income of the SLE patients with illness uncertainty scores using multivariate analysis of variance also differed significantly. (*F =* 5.578, *P* = 0.001). All results are shown in Table 2.

### Social support

The SSRS scores of patients with SLE ranged from 17 to 58, with a mean of 39.32 ± 7.43. Table 4 presented a comparison of the SSRS scores in each subscale in our study with the Chinese norms [22]. The Chinese norm referred to social support scores in healthy people as measured by Chinese experts.

**Table 4:** Comparison of Scores in each Dimension of Social Support between SLE Patients and Chinese Norm*.

Variables	SLE Patients (n = 200)	Norm (n = 307)	*P—*Value**
**Objective support**	8.85 ± 3.17	12.68 ± 3.47	<0.001
**Subjective support**	23.46 ± 4.99	23.81 ± 4.75	0.286
**Support availability**	7.01 ± 1.84	9.38 ± 2.40	<0.001
**Total**	39.32 ± 7.42	44.34 ± 8.38	<0.001

*Values are expressed as mean ± standard deviation unless otherwise specified.

**P value were calculated using independent sample *t*-test to detect the significance.

### Coping modes

A comparison of MCMQ scores in our study with the scores of general patients as reported by Shen [21] (norm) are shown in Table 5.

**Table 5:** Comparison of MCMQ scores in Patients with SLE and Patient Norm from Shen*.

Variables	SLE Patients (n = 200)	Norm (n = 701)	*P -*Value**
**Facing**	18.58 ± 3.36	19.97 ± 3.81	<0.001
**Avoiding**	15.95 ± 3.50	14.44 ± 2.97	<0.001
**Yielding**	9.30 ± 2.66	8.81 ± 3.17	<0.001

MCMQ, Medical Coping Modes Questionnaires.

*Values are expressed as mean ± standard deviation unless otherwise noted.

**P values were calculated using independent sample *t*-test to detect the significance.

### Correlation of MUIS and SSRS scores

**Fig 1:**
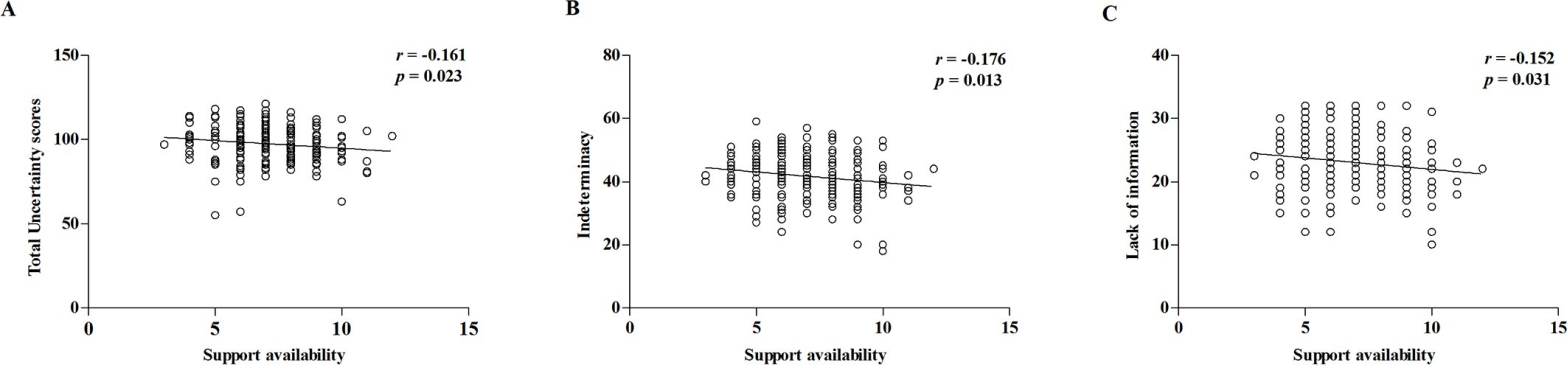
Correlation of MUIS and SSRS scores. (A): Support availability was negatively correlated with total MUIS scores of hospitalized patients with SLE. (B-C): Analysis of the sub-items in the MUIS and Social Support Scale showed a negative correlation between support availability and the indeterminacy and lack of information in the MUIS scale. The confounders as gender, marital status, educational level, disease duration, self-perceived severity of disease, payment of medicine and family income were adjusted during the analysis.

Spearman correlation analysis revealed that the total MUIS scores of patients with SLE were negatively correlated with support availability scores (Fig 1A, *r =* -0.161, *P* = 0.023). The indeterminacy and lack of information of MUIS also were negatively correlated with support availability score (Fig 1B and 1C, *r =* -0.176, -0.152, *P* = 0.013, 0.031), suggesting an increased support availability, and decreased illness uncertainty in patients with SLE.

### Correlation of MUIS and MCMQ scores

**Fig 2:**
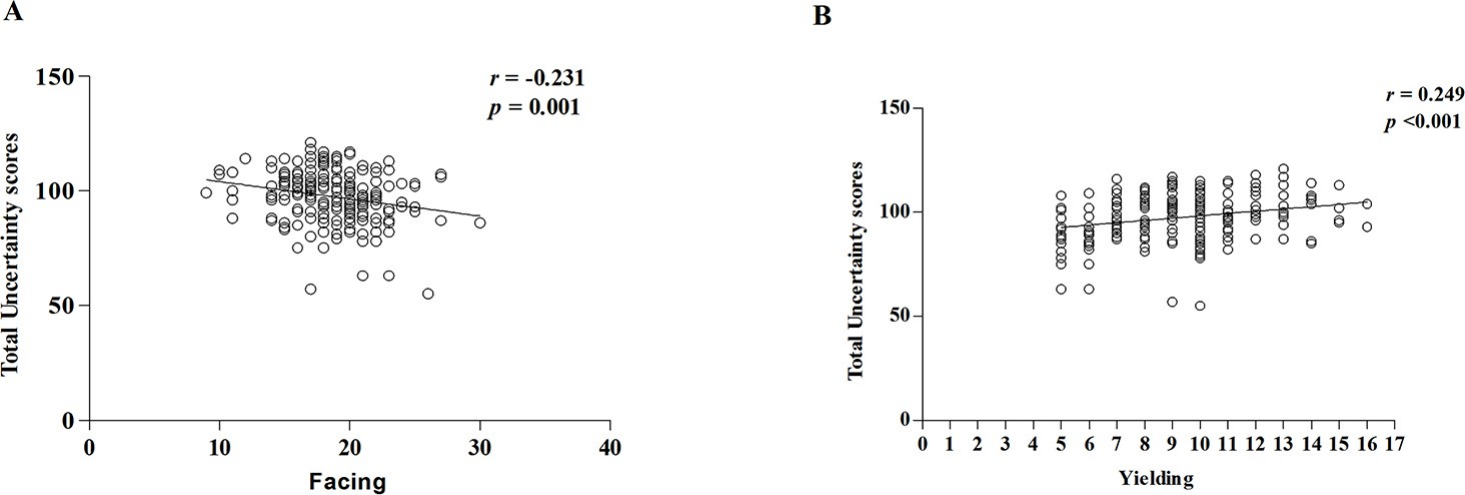
Correlation of MUIS and MCMQ scores. (A): Total uncertainty scores were negatively correlated with the coping modes of facing up the disease in the hospitalized patients with SLE. (B): Total uncertainty scores were positively correlated with the yielding coping mode. The confounders as gender, marital status, educational level, disease duration, self-perceived severity of disease, payment of medicine and family income were adjusted during the analysis.

Spearman correlation analysis indicated that MUIS scores were negatively correlated with the coping modes of facing up the disease (Fig 2A, *r = -* 0.231, *P* = 0.001), and positively correlated with the yielding coping mode (Fig 2B, *r =* 0.249, *P* <0.001). In other words, illness uncertainty was increased in patients who tended to yield as a coping mode, but decreased in many patients who were engaged in facing up the disease.

## Discussion

### Main findings

Our findings demonstrated that the illness uncertainty of 200 hospitalized SLE patients under investigation was in moderate level. Unpredictable disease process and prognosis as well as unclear symptoms are the main sources of illness uncertainty, and educational level and family income are the major influencing factors. Our study indicated that most of the patients with high illness uncertainty were those with junior-senior high school education level and monthly income below 3000 yuan.

The 200 hospitalized SLE patients had lower social support score than that of Chinese healthy norm. The total scores of illness uncertainty, the scores of indeterminacy and lock of information were negatively correlated with support availability, suggesting that the hospitalized SLE patients had less social support. Thus, improved social support can possibly reduce the illness uncertainty of SLE patients.

Compared with general patients, hospitalized SLE patients had lower scores of facing, but higher scores of avoiding and yielding, indicating that the patients could not actively face the disease, but often show avoidance and yieldingness in response to the disease. Illness uncertainty was positively correlated with scores of yielding, but negatively correlated with facing. This suggested that there was a high possibility of hospitalized SLE patients to yielding when the scores of illness uncertainty were higher, and highly possible to facing when the scores of illness uncertainty were lower.

### Comparison to other studies

Illness uncertainty not only interfere the ability of seeking for relevant information in patients, but also influence the appropriate decision making under health care. Meanwhile, it may also cause anxiety in patients, influencing their mental adaptive ability, leading to decrease in quality of life, level of hope and disease response capacity, and further influencing rehabilitation process and quality of life [19,23]. The average score of illness uncertainty among the 200 SLE patients was 97.60±11.24, which was in moderate level. In the 4 dimensions, the scores of unpredictability and indeterminacy were relatively high, and scores of lack of information and complexity were relatively low, and these results were in line with Xu et al.'s study from China [15].

In our study, the total score of social support in SLE patients was lower than that of Chinese healthy norm, which was consistent with the results of previous studies [6, 18]. This might be due to the low family income in most of the SLE patients' families. Our study indicated that the families of SLE patients' with total monthly income lower than 4000 yuan accounted for 74%, the ratio of patients under new rural co-operative medical system and self-supporting was 49%, and the medical reimbursement ratio of patients under new rural co-operative medical system during hospitalization was 50%. Some of the therapeutic drugs belong to the limited medical insurance reimbursement. SLE is a rheuma immune systemic chronic disease, and so the patients need long-term persistent medication. The economic support from family and society are limited, and patients cannot persistently afford the huge medical costs, leading to the decreased objective support. SLE was highly evident in female population of child-bearing period, which remains important stage for most of the patients attending school, trying to get a job and planning to build a family, and also the key stage for those who need to take family responsibility. However, the disease conditions of these patients are chronic and persistent, and so, long-term medication is needed. Thus, the patients had no normal ability of working, living or giving birth to babies, the physical and social function is limited, and the economic and family status were also reduced [24,25]. Furthermore, our study included SLE patients with high school education or below, accounting for 66%, and so, the approaches of looking for help were limited. The feeling of control on live and disease-related issues was reduced, so that they could not actively ask for help from social support and fully use the social resource, leading to the decrease in objective support and support availability. Mishel [26] believed that social support was one of the important factors that predict the illness uncertainty, and social support could directly influence the indeterminacy, complexity and unpredictability of illness uncertainty factors. Our study showed that utilization of support had negative correlation with illness uncertainty in hospitalized SLE patients. Although our results were not consistent with Mishel's study, similar results were shown in other diseases, such as cancer [27] and diabetes [28], indicating that it was associated with population and racial differences. Results of Wei et al [29] study also proved that good social support and its utilization degree could help decrease the illness uncertainty of patients. He et al. [20] study proved that the higher the support availability was, the lower the illness uncertainty would be.

Compared with Shen et al.'s study [21], the score of active facing disease of the hospitalized SLE patients was lower, but the scores of avoiding and yielding were significantly increased. The possible reason for this is that SLE is a chronic and persistent disease and its clinical course is usually unpredictable, with exacerbations and periods of remission. The mental health in SLE appeared to be influenced by perceived stress, disease duration, disease activity and cumulative organ damage [30]. Depression and anxiety were common in "non-NPSLE" [31]. During the long-term therapy of the disease, there lacked understanding and confidence, and hence, the patients could not face the influence of disease and therapy on study, work and live correctly. Especially under weak self-willpower or insufficient external support, patients often give up on themselves, bow to the inevitable or unwillingly face the reality. Furthermore, they may accept the negative influence subconsciously to seek the way out of disease, when they are unable to find a way out [26]. Our study showed that illness uncertainty had negative correlation with the coping modes of facing, but had positive correlation with yielding coping mode, which was in line with the results of Huang et al.'s study [32]. This suggested that the illness uncertainty might decrease if the SLE patients actively face the reality, communicate with medical staff, and obtain the disease-related knowledge by various ways. If the patients lose confidence on their disease and health, the yieldingness to the disease, and care on the self-health will decrease. They in turn will stop to look for relevant information of the disease, and become laissez-faire on the process and prognosis of the disease. Thus, the increased illness uncertainty possibly accelerates the development of the disease.

### Limitations of the study

The samples of our study were obtained from the SLE patients who were hospitalized over 1 week. Although the samples were representative of SLE, it still could not completely represent all the SLE patients. Objectively, the investigation was limited to manpower and financial resources and material resources, and so a multicentric study was not conducted. Also the sample size was limited.

### Clinical and research implications

In this study, 200 hospitalized SLE patients received illness uncertainty scale, social support rating scale and medical coping modes questionnaire. Results suggested that the illness uncertainty of the patients should receive more attention from the medical staff. During the diagnosis and treatment of the disease, the medical staff not only explore the patients by medical methods, but also pay attention to the influence of psychological, social and cultural factors on patients’ disease. For disease evaluation, illness uncertainty, social support system and coping modes should be focused on. Different patients should be helped to fully use the existing and feasible social support system. All the available support system should be mobilized to encourage the patients to expand the society associates and participate in social activities, depending on the help from family members, friends, collages, work unit and organic groups. Thus, the patients can obtain help, support, understanding and respect in every aspect, and in turn can obtain maximum social support, with reduced illness uncertainty, and improved quality of life. Patients should be guided to avoid yielding, but face the disease with positive attitude, and actively coordinate the treatment to improve therapeutic compliance. Meanwhile, it also strengthens the community building, allocates the medical staff to guide the patients' health, fully play the role of family, encourage family members to participate in the care of the patients, making them feel concern and love from various aspects. Thus, hospital care is continuously conducted to decrease the depressive emotion of the patients with disease, promote healthy behavior, build nursing services that cover physiology, psychology, society and culture, and integrates hospital and community, achieving more effective and safer therapeutic effects.

## Conclusion

In summary, a moderate level illness uncertainty is common in hospitalized patients with SLE, which in turn influences the mental adaptation ability and the quality of life. Medical staff should pay full attention to patients’ illness uncertainty, and help maximize their use of social support, as well as guide them in facing up their disease in order to reduce the uncertainty. This in turn improves the treatment compliance and quality of life of the patients.
